# Vaccine communication strategies among healthcare workers as a reflection of the Israeli Ministry of Health’s communication strategies before and after the COVID-19 pandemic

**DOI:** 10.3389/fpubh.2024.1377393

**Published:** 2024-05-23

**Authors:** Rana Hijazi, Anat Gesser-Edelsburg, Gustavo S. Mesch

**Affiliations:** ^1^The Health and Risk Communication Lab, School of Public Health, University of Haifa, Haifa, Israel; ^2^Department of Sociology, University of Haifa, Haifa, Israel

**Keywords:** vaccine hesitancy, healthcare workers, COVID-19 pandemic, health communication strategies, qualitative study, Israel, parents, public trust in the healthcare system

## Abstract

**Background:**

Healthcare workers play a central role in communicating information to the public regarding vaccines. Most of the literature has focused on healthcare workers’ hesitancy and doubts about getting the flu vaccine themselves. However, few studies have dealt with how they perceive their role in communicating information regarding vaccines, especially following the COVID-19 pandemic.

**Objectives:**

(1) To identify the communication strategies used by the Israeli Ministry of Health regarding vaccines during epidemic crises (before and after the COVID-19 pandemic); (2) To identify the communication strategies used by healthcare workers regarding vaccines before and after the COVID-19 pandemic.

**Methods:**

A qualitative study based on in-depth interviews was conducted among healthcare workers and used a semi-structured protocol as a research tool. A total of 18 healthcare workers were sampled using purposeful and snowball sampling.

**Results:**

Despite healthcare workers’ perception that there has been a decrease in trust in the Israeli Ministry of Health among the public following the COVID-19 outbreak, they still rely on the Israeli Ministry of Health as their primary source of information and use the same communication strategies (such as fear appeals and correcting information) as of the Israeli Ministry of Health to communicate with the public, healthcare providers, and other relevant stakeholders.

**Conclusion:**

Healthcare workers have been shaped by the professional socialization processes within the health system, leading to a predominant reliance on established communication strategies and informational channels. This reliance underscores the importance of evolving these methods to better engage with the public. To address this, there is a compelling need to innovate and adopt new communication techniques that emphasize effective dialogue and transparent interactions. By doing so, healthcare professionals can ensure that their outreach is not only informative but also responsive to the diverse needs and preferences of the community.

## Introduction

Health organizations during epidemics, and also when communicating information about children’s routine vaccines, have adopted several communication strategies to promote vaccination and encourage the public to get vaccinated (1, 2). This study seeks to shed light on some of the strategies employed by healthcare workers before and after the COVID-19 crisis.

Myth-busting – differentiating between facts and myths – is commonly used by health organizations. According to this approach, every piece of information that comes from other sources besides the health organization itself is labeled as a “myth,” while information that originates from the health organization itself is labeled as “fact” (3). Several studies have noted the problematic use of this strategy, which was found to result in a backfire effect (4–6); the public refused to accept this information unless it was supported by scientific evidence (7–9). In addition, repeating the “myth” by the health organizations was found to make the information more familiar and more likely to be true (6). Hence, studies conducted during the COVID-19 outbreak found that health organizations continue to use the same communication strategies of myth-busting and fear appeal strategies (2, 10).

Health organizations also widely used the fear appeal strategy during previous disease outbreaks. A *fear appeal strategy* attempts to persuade the public to adopt a specific action (such as vaccination or compliance with instructions) by arousing fear. This strategy is based on emphasizing the potential danger and harm that might result if the public does not adopt the messages’ recommendations (11). A comprehensive meta-analysis of fear appeal literature indicates that this strategy is ineffective (12). Moreover, fear appeal has also been associated with negative effects and responses such as risk denial, biased information processing, lower levels of self-efficacy, less attention, and a higher level of discomfort after being exposed to fear appeal messages during a vaccine promotion campaign (13, 14). Previous studies emphasized the apparent use of a fear appeal strategy by the Israeli Ministry of Health during the COVID-19 vaccination campaign (2). The use of this strategy was characterized by the language and tone politicians used to deliver information in the media (15, 16).

Health organizations have used these communication strategies and reached the public through the media, especially through channels such as social media in the last decade. However, the primary way of communicating with the public is still through healthcare workers, including nurses and physicians. Healthcare workers are considered the representatives of health organizations and as such play an essential role in public vaccination (17). This role includes communicating recommendations, providing information about vaccines, and vaccinating the public (18). Physicians and other healthcare providers are considered the most reliable source of information (19).

Parents perceive healthcare workers as a primary and trustworthy source of information about vaccination and vaccines (20) and play a central role in maintaining public trust in vaccination (21). Healthcare workers’ recommendations were found to be strong drivers of vaccine acceptance among the public. Therefore, they are in a position to empower parents to make an informed decision about vaccinating their children (19).

Due to the essential role of healthcare workers in the vaccination process and as a trusted source of information for parents, as well as influencing the parents’ attitudes regarding vaccination, there is a need for a better understanding of how they communicate vaccination information. This is further supported by the fact that most of the studies in the literature on healthcare workers and vaccines have focused on vaccine hesitancy, vaccine acceptance, and vaccination intention among healthcare workers (22–24). However, few studies have dealt with how healthcare workers perceive their role in communicating information regarding vaccines to the public, especially following the COVID-19 pandemic.

This study aims to (1) Identify the communication strategies used by the Israeli Ministry of Health regarding vaccines during epidemic crises (before and after the COVID-19 pandemic); (2) Identify the communication strategies used by healthcare workers regarding vaccines before and after the COVID-19 pandemic.

## Methods

### Research design and procedure

This study is based on a qualitative constructivist research method (25), which enables the researchers to study the meaning of the experience as it is perceived by the research subjects. In this study, healthcare workers themselves are used as the instrument for data collection to identify the communication strategies employed by healthcare workers and health organizations (26).

The study was approved by the Faculty of Social Welfare and Health Sciences Ethics Committee for research with human subjects at the University of Haifa (approval no. 421/17). The studies were conducted in accordance with the Israeli Medical Research Involving Human Subjects Law (1996) as the local legislation, and the requirements and guidelines set by the University of Haifa Ethics Committee. Written informed consent to participate in this study was provided by the participants.

### Sampling and data collection

In the first stage, the researchers performed a purposeful criterion sampling of healthcare workers such as family physicians, pediatricians, and nurses who are involved in the vaccination process with the public, including giving vaccines and communicating information regarding vaccines to the public. In the second stage, the researchers proceeded to perform snowball sampling. The study’s sample included 18 healthcare workers – 9 pediatricians, 1 physician, and 8 nurses from the Mother and Child Health Clinics were interviewed (Table 1). The duration of each interview was approximately half an hour.

**Table 1:** Interviewees’ sociodemographic characteristics (*N* = 18).

Variables		Frequency	Percentage (%)
Gender	Male	6	33.3
	Female	12	66.7
Age (years)	30–39	8	44.4
	40–49	5	27.8
	50–59	4	22.2
	≥60	1	5.6
Ethnicity	Jewish	6	33.3
	Arab	12	66.7
Profession	Pediatrician	9	50.0
	Physician	1	5.6
	Nurse	8	44.4
Workplace	Ministry of Health Child Centers	6	33.3
	Clalit Health Services	6	33.3
	Maccabi Health Services	1	5.6
	Leumit Health Services	3	16.7
	Meuhedet Health Services	1	5.6
	Private clinic	1	5.6
Seniority	3–9	4	22.2
	10–19	8	44.4
	20–29	2	11.1
	≥30	4	22.2

### Research tools

In-depth interviews were conducted based on a semi-structured protocol. In the first part, the questions referred to the period before the COVID-19 outbreak in Israel. The interviewees were asked questions about how they communicate the issue of vaccines to the parents, how the Israeli Ministry of Health communicates the issue of vaccines, how they deal with uncertainty, and how they correct misinformation regarding vaccines. In addition, the questionnaire included questions regarding the level of trust in the health system in Israel. The second part of the questionnaire aimed to examine whether the interviewees’ attitudes and perceptions changed after the COVID-19 outbreak in Israel. Therefore, this part included questions about the change in the level of trust in the health system in Israel, vaccine hesitancy after the COVID-19 outbreak, and how the health organizations in Israel communicated the information about the COVID-19 virus and its vaccine to the public.

### Credibility and validity

The qualitative interviews were conducted in the language preferred by the interviewee (Hebrew or Arabic) and they were audio-recorded. Then, they were transcribed verbatim, and the Arabic interviews were translated into Hebrew. The validity and reliability of this study were established based on the framework presented by Lincoln and Guba (26). These researchers suggested the term “trustworthiness” to evaluate the validity and reliability of the qualitative research. *Trustworthiness* is demonstrated through four components. The first component is credibility (comparable with internal validity) and refers to the “fit” between the participants’ views and the researchers’ presentation of the findings. Transferability (comparable with external validity) is the second component, which addresses the issue of generalizability of inquiry and the researchers’ ability to anchor the research findings in other similar cases and other relevant theories. The third component is dependability (comparable with reliability), which is achieved by ensuring that the research process is logical, traceable, and clearly documented. The last component is confirmability (comparable with objectivity or neutrality), which is concerned with establishing that the findings do not arise from the researchers’ assumptions, prejudices, interests, and motivations, but are clearly derived from the data and allow for the acceptance of the conclusions arising from the findings (27). This study achieved these components through prolonged engagement, persistent observation, peer debriefing, and audit trials (28).

### Analysis

Qualitative data were analyzed using thematic analysis (29). Specifically, we focused on text codes regarding the participants’ attitudes toward vaccination, dilemmas in communicating the information regarding vaccines to the public, the public’s perceived level of trust in the health system, and how participants perceive health organizations’ communication methods in Israel before and after the COVID-19 outbreak. These codes were then grouped into themes and sub-themes, and following the rules for inclusion in each category, relevant texts from subsequent interviews were coded.

## Results

Four main themes emerged from the analysis of the interviews (Table 2). The themes focused on the communication strategies used by healthcare workers and the Israeli Ministry of Health before and after the COVID-19 outbreak, and on how the participants perceived these strategies. In addition, the issue of trust in the Ministry of Health also emerged as a main theme.

**Table 2:** Themes and sub-themes.

Theme	Sub-themes
1.Trust in the Israeli Ministry of Health	1.1. Healthcare workers’ trust in the Ministry of Health 1.2. The public’s level of trust in the Ministry of Health before and after the COVID-19 outbreak in Israel
2.Information communication by the Israeli Ministry of Health	2.1. Fear appeals as the main strategy for communicating information 2.2. Correcting misinformation 2.3. Segmentation of the public
3.How do healthcare workers perceive the Ministry of Health’s communication strategies and its ways of conveying information to the public	3.1. The controversy regarding transparency and providing complete information 3.2. Communicating uncertainty 3.3. The Ministry of Health is the most reliable source of information for healthcare workers 3.4. Healthcare workers as role models
4.How healthcare workers perceive children’s vaccination and parents who are hesitant or anti-vaccination	4.1. Concerns following the recommendation to vaccinate children with the COVID-19 vaccine 4.2. How healthcare workers perceive anti-vaccination parents

### Trust in the Israeli Ministry of Health

Trust in the Ministry of Health emerged as a main theme in the study. The interviewees referred to (1) healthcare workers’ trust in the Ministry of Health, and (2) the public’s level of trust in the Ministry of Health before and after the COVID-19 outbreak in Israel.

#### Healthcare workers’ trust in the Ministry of Health

Seven interviewees out of 18 expressed a high level of trust in the Ministry of Health. The interviewees described the Ministry of Health as a reliable and trustworthy source of health information in general and as regards vaccine information.

“I trust all the information provided by the Ministry of Health. This information is reliable and trustworthy” (Interviewee 12).

In addition, the interviewees expressed a high level of trust in the health authorities represented by the Ministry of Health and policymakers. They claimed that policymakers always make the best decisions for public health. Their decisions are based on studies and motivated by concern and responsibility for public health and not by other hidden interests. “I am confident in the policymakers and their decisions. I trust the authorities. The decisions regarding vaccines aim to promote public health and are not motivated by hidden interests” (Interviewee 7).

#### The public’s level of trust in the Ministry of Health before and after the COVID-19 outbreak in Israel

Most interviewees referred to the change in the public’s level of trust in the Ministry of Health after the COVID-19 outbreak in Israel. Fourteen out of 18 interviewees mentioned that the public’s trust in the Ministry of Health was higher before the COVID-19 outbreak in Israel. However, following the COVID-19 outbreak in Israel, the public’s level of trust in the Ministry of Health decreased because of crisis management issues, particularly inconsistent instructions, inconsistent statistical reporting of the COVID-19 cases, political and economic motives, and using an intimidation-based communication strategy.

“The level of trust has decreased because of corruption and political and economic interests… The public doesn’t feel their personal interest is a priority of the authorities… Instead, they see that there are all kinds of other interests and things that influence the authorities' decisions such as politics, ego, power, and economic interests” (Interviewee 10).

“After the COVID-19 outbreak, the level of trust decreased. The health authorities reported inconsistent statistics regarding the number of COVID-19 cases, in general, and serious cases, in particular. The authorities scared the public, and people understood that the number of reported patients was not accurate” (Interviewee 11).

In addition, some interviewees claimed social media was the reason for the decrease in the public’s trust in the Ministry of Health after the COVID-19 outbreak. The public was exposed to anti-vaccination information and intense debate on social media regarding the COVID-19 vaccine’s effectiveness and safety.

“Before the COVID-19 outbreak, the public’s trust in the Ministry of Health was higher. Fewer people were exposed to what was written on social media and anti-vaccination messages. Some parents didn’t know that there are parents who oppose vaccines and don’t vaccinate their children” (Interviewee 14).

However, one interviewee claimed that the drop in the public’s level of trust in the Ministry of Health was a temporary situation and that the level of trust has returned to what it was before the COVID-19 outbreak.

“I think the public has already forgotten what happened during the COVID-19 outbreak, and the trust has returned to its previous level. There was no real loss of trust … It’s just that at first, we received ambiguous instructions because there were constant changes in the instructions… Almost every week, we received different instructions, and we were confused” (Interviewee 1).

### Information communication by the Israeli Ministry of Health

The interviewees described the communication strategies used by the Ministry of Health. These strategies included fear appeals and correcting misinformation. In addition, they described the information delivered by the Ministry of Health as being uniform, without adapting the information to the different public groups.

#### Fear appeals as the main strategy for communicating information

Most interviewees (15 out of 18) claimed the Ministry of Health used and still uses fear appeal as the primary strategy to promote vaccination before and after the COVID-19 outbreak. As described by the interviewees, this strategy is based on persuasive messages that attempt to arouse fear among the public to promote routine vaccines and the COVID-19 vaccine. These messages emphasized the severity of the disease, the risk of not being vaccinated, reported statistics of infected cases and deaths, and sanctions against those who are not vaccinated and fail to comply with the health authorities’ recommendations. They described this strategy as effectively motivating the public to vaccinate and claimed that imposing sanctions – such as preventing unvaccinated children from entering kindergarten – against unvaccinated individuals is legitimate.

“The act of not letting an unvaccinated child into kindergarten is completely acceptable. I think that unvaccinated children should not be protected indirectly because other children are vaccinated” (Interviewee 8).

“The public got the vaccine because the Ministry of Health scared them and forced them to get vaccinated. The Ministry of Health described the virus as very contagious and said the disease was severe” (Interviewee 2).

#### Correcting misinformation

Healthcare workers and health organizations play a critical role in addressing and correcting misinformation. Thirteen interviewees described the methods by which they and the health organizations correct misinformation. For example, healthcare workers may face misinformation regarding vaccines during their appointments with parents. According to the interviewees, healthcare workers’ ways of correcting information included explaining the importance of vaccination in preventing infectious diseases to the parents, assessing the risks and benefits of vaccines, and explaining that clinical trials have been conducted to approve vaccines just like other medicines.

“I give an example of the medicines they receive. Furthermore, I explain that both these medicines and vaccines have undergone clinical trials. Moreover, I also present the statistics and how people in the past died before vaccines were invented” (Interviewee 4).

In addition, the interviewees dealt with misinformation by referring parents to the Ministry of Health website for more information or giving them medical information leaflets provided by the Ministry of Health. Another way to deal with the misinformation suggested by the interviewees is to rely on their rich experience and say that this information needs to be corrected.

“I refer parents to the Ministry of Health website and explain that I’ve been working here for years and that they should trust me. I also try to explain more about the risks and benefits of vaccines” (Interviewee 8).

“I explain that during the 20 years that I’ve worked as a Tipat-Halav (Mother and Child Health Clinics) nurse, I’ve never seen any child who suffered from unusual symptoms or side effects such as those which you, the parent, mentioned … I say that even if it’s true, then it occurs among a very small percentage of children” (Interviewee 2).

Two interviewees claimed that they would search for studies addressing and correcting misinformation from other sources of information besides the Ministry of Health, such as studies conducted in other countries. In addition, one interviewee also suggested referring parents to reliable information-seeking sources.

“To deal with misinformation, I search for other studies carried out in Israel and abroad, and I send these studies to the parents or even tell them where to search for reliable information” (Interviewee 7).

The interviewees also mentioned that health organizations correct misinformation by presenting the misinformation and its correction. To correct misinformation, health organizations used healthcare workers who appeared in the media or posted posts on social media mentioning fake news.

“Healthcare workers and experts appeared in the media to correct misinformation” (Interviewee 18).

“The Ministry of Health started to correct the misinformation through social media during the COVID-19 outbreak by mentioning the misinformation and labeling it as false or fake” (Interviewee 14).

#### Segmentation of the public

The way the Ministry of Health communicates information in general, and on the topic of vaccination in particular, emerged as a theme from the interviews. Fifteen interviewees (out of 18) described the information provided by the Ministry of Health as uniform and addressed to the broad public. This means the transmitted information is not adapted to the different groups which comprise the general public.

“I think the Ministry of Health provides everyone with the same information, but everyone deals with the information differently” (Interviewee 15).

### How healthcare workers perceive the Ministry’s of Health communication strategies and its ways of conveying information to the public

This main theme consisted of four sub-themes: (1) the controversy regarding transparency and providing complete information; (2) communicating uncertainty; (3) the Ministry of Health is the most reliable source of information for healthcare workers; and (4) healthcare workers as role models.

#### The controversy regarding transparency and providing complete information

On the one hand, four interviewees out of 18 claimed that the Ministry of Health does not provide the public with complete and transparent information. In addition, they mentioned that the Ministry of Health needs to be transparent and provide complete and accurate information to the public, so they can make informed decisions regarding their health. Moreover, some interviewees argued that not providing complete information underestimates the public’s intelligence and that the health authorities perceive the public as being unable to deal with all the information and make the best decisions regarding their health.

“The Ministry of Health certainly does not provide complete information to the public. Instead, it provides the information it thinks the public should know. The Ministry of Health doesn’t think the public is intelligent enough to make their own health decisions. The authorities perceive the public as lacking the ability to read, understand, and ask questions. You have a doctor, do what he tells you. Today, it is no longer like that; some patients know more about their diseases than their doctors because they have access to the information. I believe underestimating the public’s intelligence is not good” (Interviewee 10).

On the other hand, six interviewees out of 18 suggested that the Ministry of Health should not provide complete information to the public. They also claimed that the public could not deal with complete information and that providing it might create confusion among the public. Therefore, the Ministry of Health should make public health decisions and provide information that promotes vaccination or motivates the public to make the decision recommended by the Ministry of Health.

“I think providing complete information is wrong because the patient should play the role of the patient, and the doctor should play the role of the doctor. The patient should not cross this border… The information should be conveyed assertively, and in a goal-oriented manner… and the goal should be to promote vaccination” (Interviewee 4).

#### Communicating uncertainty

Communicating uncertainty to the public was examined through nine interviewees. On the one hand, seven interviewees mentioned that the public could not handle or deal with uncertainty and that communicating uncertainty might generate confusion and “hysteria” among the public. Therefore, health organizations should provide only specific information.

“I think the public cannot deal with uncertainty. In addition, communicating uncertainty may generate confusion. It also requires a lot of explanation and effort, which burdens the health system” (Interviewee 4).

On the other hand, two interviewees mentioned that the public wants to receive complete and transparent information, including uncertain information. Moreover, it is the public’s right to receive complete information. Therefore, health organizations need to practice transparent communication.

“The public should receive all the information, including uncertainty … there is no such thing as hiding information. The information should be transparent… I think the Ministry of Health does not communicate all the information” (Interviewee 6).

#### The Ministry of Health is the most reliable source of information for healthcare workers

Thirteen interviewees out of 18 mentioned that for them the only source of information regarding vaccines is the Ministry of Health. Therefore, they only share information from the Ministry of Health with parents or on social media. Moreover, some mentioned that they do not know of any other sources of information besides the Ministry of Health that are reliable and trustworthy.

“I’m not aware of any other sources besides the Ministry of Health. We get the information only from the Ministry of Health” (Interviewee 12).

"I only receive information from the Ministry of Health and share it on social networks as well” (Interviewee 13).

In addition, some interviewees mentioned that parents who ask for more information from other sources are referred to websites recommended by the Ministry of Health.

“We give parents who ask for more information a website we received in lectures for Tipat-Halav nurses. This is a website recommended by the Ministry of Health … We refer concerned parents to this site along with those who say they prefer to postpone getting the vaccine for another month…” (Interviewee 8).

#### Healthcare workers as role models

Six interviewees out of 18 claimed they try to encourage hesitant and anti-vaccination parents to vaccinate their children by mentioning that they themselves, as well as their children, are vaccinated.

“In addition to explaining and talking about studies, I would tell the parents about myself, that I am vaccinated, and that my children are vaccinated. This convinced most of them to vaccinate their children” (Interviewee 15).

However, three interviewees out of 18 described the difficulty of promoting some vaccines, such as the COVID-19 vaccine, the Polio vaccine, and the Rotavirus vaccine. They said that because they consider themselves role models, it is difficult to recommend hesitant and anti-vaccination parents to vaccinate their children when they, themselves, do not vaccinate their children with these vaccines or believe that these vaccines are effective and safe.

"It was difficult for me to recommend the Corona vaccine for both adults and children. I had concerns because I know several people who suffered from side effects from the vaccine” (Interviewee 14).

“I have concerns regarding the safety of the Polio vaccine. Only two of my children were vaccinated with this vaccine. Therefore, when a hesitant parent asks me what I did, it’s hard for me to tell them that I didn’t vaccinate all my children because we are role models. Sometimes, I say that it doesn’t matter what I did. Moreover, sometimes, I say that I did vaccinate my children … Regarding the Rotavirus vaccine, it’s hard for me to encourage hesitant parents to vaccinate their baby because I know that the baby may suffer from side effects and pain” (Interviewee 9).

### How healthcare workers perceive children’s vaccination and parents who are hesitant or anti-vaccination

The interviewees described their concerns and doubts regarding vaccinating children with the COVID-19 vaccine. In addition, they mentioned in this main theme how they perceive and communicate with hesitant and anti-vaccination parents.

#### Concerns following the recommendation to vaccinate children with the COVID-19 vaccine

The interviewees (nine out of 18) expressed concerns about the recommendation to vaccinate children with the COVID-19 vaccine. They claimed that authorities were supposed not to rush recommending vaccinating children because the COVID-19 vaccine was new. In addition to fewer severe reported cases of Corona disease among children, more information was available regarding short-term side effects, and long-term side effects were still being determined. Therefore, they were not confident about recommending the COVID-19 vaccine for children.

“Regarding the COVID-19 vaccine, my family and I were vaccinated, but regarding children's vaccinations, I would wait with that and not rush to vaccinate. Because children are not at risk, especially healthy children” (Interviewee 12).

“It was tough for me to recommend the COVID-19 vaccine because the vaccine is new and its side effects are still unknown” (Interviewee 11).

#### How healthcare workers perceive anti-vaccination parents

Eleven interviewees described how they perceive anti-vaccination parents. Most of them (9 out of 11) think that anti-vaccination parents oppose vaccines because of their ideological perceptions and their opposing attitudes toward the government, or they think the vaccination process is motivated by hidden interests. In addition, anti-vaccination parents’ attitudes rely on the information they receive from social media or rumors that are not evidence-based. Therefore, these healthcare workers think it is not worth trying to conduct a dialogue with these parents because they will not be convinced.

“They decide not to vaccinate without scientific proof and only because they heard that the vaccine could cause harm from their neighbors or someone who knows nothing … their information is not scientifically based” (Interviewee 11).

“The opposition to the government is expressed by their opposition to the vaccines… At the same time, many groups with agendas publish fake news and misinformation on social media… I don’t have the time or the patience to try and talk with them” (Interviewee 5).

However, three interviewees mentioned that there are anti-vaccination parents whose attitudes are based on scientific information such as studies and articles. In addition, they described these parents as highly educated.

“I was surprised to find that some anti-vaccination parents are highly educated. Some are physicians and refuse vaccines out of knowledge. For example, they cite side effects listed in the American leaflet and not in the Israeli leaflet, and they cite scientific studies and quotes from expert doctors from abroad” (Interviewee 16).

## Discussion

The literature has indicated the unique and essential role of healthcare workers in mediating the public’s trust in health authorities and policymakers. Previous studies have found that trust in the healthcare system is essential to public compliance with the authorities’ recommendations and guidelines during epidemics (30, 31). The public’s trust in the healthcare system includes trust in the health authorities, healthcare workers, and policymakers (32). Trust was also found to influence the public’s acceptance of vaccines. Higher trust in the healthcare system is associated with lower barriers and higher acceptance of vaccines (33, 34). The COVID-19 outbreak has elucidated the importance of establishing trust in the health authorities among the public. Recent studies have emphasized the importance of trust in the healthcare system and its association with compliance with both the COVID-19 vaccine and the authorities’ recommendations (35–40). In this study, healthcare workers reported a decrease in the public’s trust in the health authorities following the COVID-19 outbreak in Israel. However, despite the decrease in the public’s trust in the health authorities, the study findings show a high level of trust in the health authorities among healthcare workers during the COVID-19 outbreak in Israel.

An essential aspect related to healthcare workers’ trust in this study is the fact that many perceive the health authorities as their primary and only source of credible and reliable information. Previous studies have found that although the public draws upon various sources of information, such as social media (41), healthcare workers are still perceived as a primary and trustworthy source of information about vaccination (20). As part of the healthcare workers’ role, they are required to provide information to the public, address their concerns, and answer their questions. Therefore, healthcare workers need to have up-to-date and comprehensive information (42) to serve as a reliable source for the public (43).

The same ambivalent approach represents the rest of the participants’ attitudes in this study. For example, most of the participants claimed that the Ministry of Health uses a fear appeal strategy to communicate with the public. They stated that the Israeli Ministry of Health uses fear-based messages to promote vaccinations by emphasizing disease severity, risks of being unvaccinated, infection/death statistics, and sanctions for non-compliance. Participants viewed this approach and restricting kindergarten access for the unvaccinated as effective and legitimate motivators. Consistently, previous studies found that fear appeal strategy was widely employed in COVID-19 public health messages to promote preventive behaviors by highlighting illness or death risks (44, 45). However, overreliance on fear risks unintended consequences (46). While fear can motivate if applied judiciously, balancing severity, susceptibility, self-efficacy and response efficacy is complex (47). For instance, a Kenyan case study found job/income loss threats promoted hand sanitizing and masking among taxi drivers (48). In addition, previous studies found that sanction risk had no significant effect on compliance (49, 50).

Attitudes of the participants also diverged regarding transparency. Some stated that the Ministry of Health should provide complete information and communicate with transparency. On the other hand, still, other healthcare workers in this study believe the communication strategies used by the Ministry of Health are effective and think the Ministry of Health should provide the public with only partial information.

The same holds true regarding their perceptions about vaccinating children with the COVID-19 vaccine. Although they reported having concerns and doubts regarding the vaccine’s safety and admitted they did not have sufficient information regarding the vaccine’s side effects among children, they still communicated the importance of vaccinating children and recommended this vaccine for children. In addition, they said they believed the health authorities had successfully managed the COVID-19 crisis. Contrary to these findings, a recent study showed that Israeli health professionals expressed varied levels of trust in the authorities and a moderate level of trust in policy during the first wave of COVID-19 (51).

The findings indicate that despite health workers’ reservations about a variety of issues (the use of fear appeal, child vaccinations, etc.), they still perceive the Ministry of Health as a reliable and central source of information and used the same strategies to convey information to the public during COVID-19 pandemic. These findings may be explained by the socialization of healthcare workers, which is defined as “a process in which people learn to adapt to values, skills, points of view, norms, and knowledge needed for belonging to a community, group, or organization” (52).

The inconsistent findings in this study regarding the high level of trust among healthcare workers, their perception of the public’s decreased level of trust in the Ministry of Health, and their hesitant attitudes regarding giving children the COVID-19 vaccine may be explained by cognitive dissonance theory. This theory claims that inconsistency between attitudes, beliefs, and behaviors may create cognitive dissonance and a lack of harmony accompanied by psychological stress. Therefore, people seek psychological consistency by resolving the dissonance and aligning their cognitions with their actions. Blindly trusting whatever they want to believe or avoiding contradictory information are ways to establish psychological consistency and reduce the magnitude of the dissonance (53, 54). For example, recent research found that inducing cognitive dissonance improved COVID-19 safety compliance and vaccination attempts versus controls (55). Concerning the findings of this study, cognitive dissonance may provide a useful framework for several reasons. First, it directly links the inconsistencies observed between trust in the health authorities and attitudes toward vaccination. Second, seeking consistency to resolve dissonance fits with healthcare workers’ tendency to maintain trust and recommend the COVID-19 vaccine despite doubts raised. However, cognitive dissonance theory faced limitations as alternative theoretical frameworks and models were proposed to account for various dissonance-related findings (56–59). For instance, Bem (60, 61) self-perception theory and Tedeschi et al. (62) impression management theory aimed to explain dissonance effects observed in classic studies like Festinger and Carlsmith (63). Additionally, early research by Heine and Lehman (64) suggested that cultural factors may moderate experiences of dissonance. Social cognition theories and theories about motivational processes were also put forth as alternative explanatory perspectives (65, 66). Some experimental designs further challenged certain specific aspects of cognitive dissonance theory (67, 68). Therefore, other interpretations may be possible, and cognitive dissonance may not fully account for the complex factors influencing attitudes and behaviors. With these limitations in mind, cognitive dissonance theory still offers one approach for comprehending how healthcare workers reconcile their conflicting attitudes and perceptions to establish psychological consistency and reduce dissonance through vaccine recommendations and trust in authorities.

Another theory that may explain the study findings is Heider’s balance theory, which reinforces the cognitive dissonance theory. This theory is based on a triadic model where a balance must exist among the three subjects involved. Therefore, the individual tends to seek modifications to maintain cognitive and emotional harmony and balance (54, 69, 70). According to this theory, healthcare workers in this study seek to maintain a balance between their trust in the science of vaccines, their trust in the Ministry of Health (which promotes vaccines), and their communication strategies and recommendations for the COVID-19 vaccine. Thus, a higher level of trust among healthcare workers in the science of vaccines leads to a higher level of trust in the Ministry of Health (which promotes vaccines), eventually resulting in behavior that reflects vaccination recommendations and adopts the same communication strategies as the Ministry of Health.

## Study limitations

This is a qualitative study that does not profess to include a representative sample of healthcare workers. However, the strength of the qualitative study lies in the richness and depth of the collected data. A clear limitation is the small sample size (only 18 healthcare workers were interviewed). Consequently, the results cannot be generalized to the entire population of Israeli healthcare workers. It is important to note, though, that thematic saturation was achieved within the analysis. In addition, the sample consisted of specific categories of healthcare workers (pediatricians, a physician, and nurses), who primarily work at child health centers that belong to the Ministry of Health and health organizations. As such, it is possible that the attitudes and perceptions shared may not fully encompass the diversity of views among Israel’s broader healthcare community, which includes a more extensive array of professional roles and specializations. Therefore, future studies should include other healthcare workers besides the ones in the study sample. Further studies should also be conducted to evaluate the healthcare workers’ trust in the health authorities, due to a dearth of these studies in the literature. It is important to note that this study was partly conducted before the COVID-19 outbreak and resumed after the COVID-19 vaccination campaign. Therefore, it aimed to address the change in attitudes and perceptions among healthcare workers regarding the communication of the vaccine issue. Thus, follow-up studies on healthcare workers’ communication strategies should be conducted.

## Conclusion

In summary, the study’s findings indicate that healthcare workers have undergone professional socialization by the health system. Despite healthcare workers’ perception that there has been a decrease in the public’s trust in the Ministry of Health following the COVID-19 outbreak, the workers, themselves, continue to adopt the same communication strategies as the health authorities. Therefore, to increase the public’s trust in both the healthcare system and in healthcare workers, multifaceted approaches and policies are recommended. Health organizations, authorities, and healthcare workers need to change their communication strategies to regain public trust. This includes transparently providing complete information, encouraging open dialogue to address public concerns and fears, and empowering healthcare workers to engage patients through dedicated discussion time and communication training. Health authorities and workers should also collaborate on unified messaging campaigns to enhance credibility, while tailoring outreach efforts to acknowledge the diversity of public perspectives across different demographic groups. It is important to note that while these recommendations are based on the findings from this study, the limited generalizability of the study should be considered.

## Data availability statement

The raw data supporting the conclusions of this article will be made available by the authors, without undue reservation.

## Ethics statement

The studies involving humans were approved by the Faculty of Social Welfare and Health Sciences Ethics Committee for research with human subjects at the University of Haifa (approval no. 421/17). The studies were conducted in accordance with the local legislation and institutional requirements. The participants provided their written informed consent to participate in this study.

## Author contributions

RH: Conceptualization, Data curation, Formal analysis, Investigation, Validation, Writing – original draft, Writing – review & editing. AG-E: Conceptualization, Formal analysis, Supervision, Validation, Writing – original draft, Writing – review & editing. GM: Supervision, Writing – review & editing.
